# A mobile app using therapeutic exercise and education for self-management in patients with hand rheumatoid arthritis: a randomized controlled trial protocol

**DOI:** 10.1186/s13063-020-04713-4

**Published:** 2020-09-10

**Authors:** Pablo Rodríguez-Sánchez-Laulhé, Luis Gabriel Luque-Romero, Jesús Blanquero, Alejandro Suero-Pineda, Ángela Biscarri-Carbonero, Francisco José Barrero-García, Alberto Marcos Heredia-Rizo

**Affiliations:** 1 Andalusian Public Foundation for the Management of Health Research of Seville FISEVI, Seville, Spain; 2 Research Unit, Distrito Sanitario Aljarafe-Sevilla Norte, Servicio Andaluz de Salud, Seville, Spain; 3 Normal and Pathological Cytology and Histology Department, University of Seville, Seville, Spain; 4 Physiotherapy Department, Faculty of Nursing, Physiotherapy and Podiatry, University of Seville, Seville, Spain

**Keywords:** Education, E-health, Exercise therapy, Mobile applications, Protocol, Randomized controlled trial, Rheumatoid arthritis, Self-management, Tele-rehabilitation

## Abstract

**Background:**

Therapeutic exercise is a safe and cost-effective approach to alleviate hand rheumatoid arthritis (RA)-related symptoms. This study aims to investigate the differences in self-management between a smartphone app (CareHand), using hand exercises and educational advices, compared with a standard approach, on hand overall function, pain intensity, stiffness, and grip and pinch strength in patients with hand RA.

**Methods:**

The project is a prospective, longitudinal, superiority, randomized controlled trial. Fifty-eight participants with hand RA will be randomly assigned into an experimental group (CareHand app) or a control group (conventional treatment). Control intervention involves a paper sheet with exercises and recommendations, and the experimental group includes the use of a smartphone app, which provides individualized exercise programs, self-management, and educational strategies to promote adherence to treatment. Both intervention protocols will last for 3 months. The principal investigator will conduct an educational session at baseline for all participants. Primary outcome comprises the overall hand function, assessed with the Michigan Hand Outcome Questionnaire (MHQ). Secondary outcomes include self-reported functional ability with the Quick DASH questionnaire, self-reported pain intensity and morning stiffness using a Visual Analogue Scale (VAS), and hand grip and pinch strength (dynamometer). Outcome measures will be collected at baseline, and at 1 month and 3-month follow-up.

**Discussion:**

This study will evaluate the effectiveness of a tele-rehabilitation tool, which uses exercise and self-management strategies, compared to a conventional approach, in patients with hand RA. The smartphone app will allow to monitor the patient’s status and to enhance patient-therapist communication. Some limitations may be related to the short follow-up duration and the lack of evaluation of psychosocial factors. Overall, this new way of promoting long-term effects in patients with a chronic rheumatic disease could be feasible and easy to implement in daily life clinical practice and current musculoskeletal care.

**Trial registration:**

ClinicalTrials.gov NCT04263974. Registered on 7 March 2020. Date of last update 15 April 2020. Ethics committee code: PI_RH_2018.

## Administrative information

Note: the numbers in curly brackets in this protocol refer to SPIRIT checklist item numbers. The order of the items has been modified to group similar items (see http://www.equator-network.org/reporting-guidelines/spirit-2013-statement-defining-standard-protocol-items-for-clinical-trials/).

**Table:** 

Title {1}	A mobile app using therapeutic exercise and education for self-management in patients with hand rheumatoid arthritis. A randomized controlled trial protocol. Trial Acronym: CH_HAR
Trial registration {2a and 2b}.	Effectiveness of an exercise program and education through a mobile application for the management of patients with hand osteoarthritis and rheumatoid arthritis Trial identifier: NCT04263974
Protocol version {3}	Initial release: 7 March 2020 Last update: 15 April 2020
Funding {4}	Andalusian Public Foundation Progreso y Salud, Health and Families Council of the Andalusian Government, Spain (funding reference number AP-0149-2017)
Author details {5a}	SPIRIT guidance: Affiliations of protocol contributors. Pablo Rodríguez-Sánchez-Laulhé ^1^ Luis Gabriel Luque-Romero ^2,3^ Jesús Blanquero ^4^ Alejandro Suero-Pineda ^4^ Ángela Biscarri-Carbonero ^2^ Francisco José Barrero-García ^2^ Alberto Marcos Heredia-Rizo ^4^ ^1^ Andalusian Public Foundation for the Management of Health Research of Seville FISEVI, Seville, Spain ^2^ Research Unit, Distrito Sanitario Aljarafe-Sevilla Norte, Servicio Andaluz de Salud, Seville, Spain ^3^ Normal and Pathological Cytology and Histology Department. University of Seville, Spain ^4^ Physiotherapy Department, Faculty of Nursing, Physiotherapy and Podiatry, University of Seville, Spain
Name and contact information for the trial sponsor {5b}	Andalusian Public Foundation for Heath Research Management in Seville Dr. José Cañón Campos, Managing Director. Fundación Pública Andaluza para la Gestión de la Investigación en Salud de Sevilla, Hospital Universitario Virgen del Rocío. Edificio de Laboratorios, 6ª Planta. Avenida Manuel Siurot, s/n, 41013, Sevilla, Spain. e-mail: jcanon-ibis@us.es, Phone: +34 955 012 820, Cell phone: +34 677 90 42 64, Fax: +34 955 013 292
Role of sponsor {5c}	Public Funding entity The funding agency financed and supervised the execution of the project, with no further involvement in the design of the study, the collection and analysis of data or the interpretation of results.

## Introduction

### Background and rationale {6a}

Rheumatoid arthritis (RA) is a chronic autoimmune disease [1] and the most common inflammatory polyarthritis [2]. Overall, the prevalence rate of RA is about 1.1% of the adult population in Europe and North America [3], with lower occurrence in Mediterranean countries, e.g., 0.5% in Spain [4]. The exact cause of AR is unknown, although genetic, epigenetic, and environmental conditions may contribute to its development [1, 5], and help to explain the high variability in geographic presentation.

Up to 90% of patients with RA report wrist or hand problems, especially metacarpophalangeal (MCP) and proximal interphalangeal (PIP) joints [6], with symptoms such as pain, swelling, decreased mobility [7], loss of muscle mass [8], hand deformities, reduced strength [9], and stiffness [10], which causes impaired function and social participation [11]. This leads to a huge socioeconomic burden, higher than that of other non-communicable diseases [12], with an estimated annual loss of productivity of 11.500$ per person [13], and an increased 50% risk of unemployment [14].

The clinical management of hand RA includes pharmacological and non-pharmacological treatments that aim to prevent or control joint damage and RA-related disability, especially overall hand function and pain [15, 16]. The target of currently available medication for RA, e.g., non-steroidal anti-inflammatory drugs, glucocorticoids, and disease-modifying antirheumatic drugs (DMARDs), is remission or low disease activity [17], and to reduce hand symptoms and deformities [18]. However, the use of these drugs increases the direct healthcare costs by 300% [19]; hence, the cost-effectiveness of this approach is controversial [17]. In addition, muscle function is not always directly improved even when the disease is controlled [18]. Among non-pharmacological interventions, hand exercise programs are safe and cost-effective to alleviate symptoms in patients with RA [12] and may even enhance the effects of medication [20]. Therapeutic exercise for RA has demonstrated to be positive for hand function, although uncertainty remains about its impact on pain and strength [16]. Yet, there is evidence of a low rate of adherence to prescribed exercises in people with upper limb conditions [21]. Further studies of high methodological quality with hand function as the main outcome and the use of strategies to increase adherence are warranted [16].

Several approaches to improve adherence and compliance to treatment have been formerly proposed, e.g., promoting self-efficacy with exercises, clarifying and solving the main barriers to physical activity, using an exercise diary with pictures and explanations, establishing feasible objectives along with verbal and written contracting [18], monitoring patients with  phone calls [22], and raising awareness of the associated risks and benefits of a concrete health behavior [23]. It has been suggested that the use of smartphone applications has a great potential to achieve these goals [18]. In fact, the combination of smartphone apps with healthcare professionals’ intervention to promote self-management may improve adherence, participation, and long-term efficacy [24–26]. Technology implantation has a key role in current practice for the control and treatment of RA, and to enhance patients’ satisfaction and general wellness [26].

CareHand is an app specifically aimed at providing home exercise programs and educational advices about RA and pain management for patients with hand RA. This app records patients’ symptoms and exercise progression to adjust the therapeutic program, which helps to increase self-management [26], and participation and compliance to the treatment protocol [11]. The app has been developed by and under the supervision of different healthcare professionals, e.g., physicians, physiotherapists, and occupational therapists, to follow the current evidence-based guidelines for the treatment of patients with hand osteoarthritis or RA.

## Objectives {7}

The aim of this project is to investigate the differences in self-management using a smartphone app (CareHand), consisting of hand exercises and educational advices, compared with a standard approach (home exercise program on paper), on hand overall function, self-reported pain intensity and stiffness, and grip and pinch strength in patients with hand RA.

We hypothesize that the use of the smartphone app, compared to the exercise program on paper, can provide greater positive impact on hand function, pain, stiffness, and strength, immediately after the intervention protocol and in a 3-month follow-up.

## Trial design {8}

**Fig. 1:**
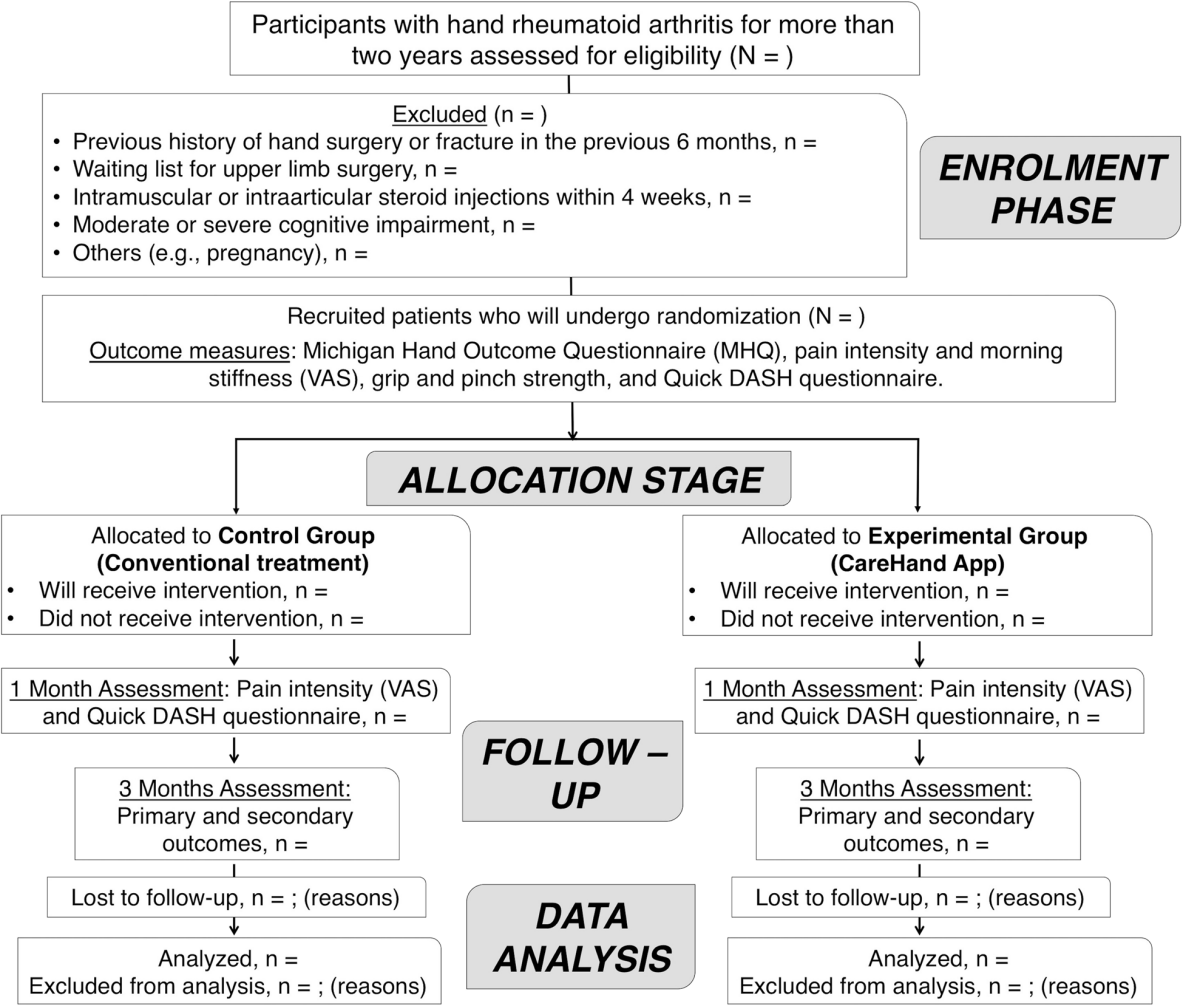
CONSORT flow diagram template

A single-blinded, parallel, two-arm group randomized, controlled, superiority trial will be conducted. The random sequence, with a 1:1 allocation of participants in the study groups, has been generated using an external website. The outcome data will be collected from a paper form or via telephone by a research assistant who is blinded to the treatment arm. The study protocol received the approval of the Research Ethics Committee of Virgen del Rocio and Virgen Macarena University Hospitals, Seville, Spain (code number: PI_RH_2018), and has been registered in ClinicalTrials.gov with registration number NCT04263974. Figure 1 lists the flowchart of the study design.

## Methods: participants, interventions, and outcomes

### Study setting {9}

Eligible participants will be selected from the database of the Northern Seville and Aljarafe District, Andalusian Health Service, Seville, Spain. Recruitment, data collection, and educational sessions will be conducted at two community health centers located in Camas and Sanlúcar la Mayor, both villages in the Seville province, Spain. Data collection training will be carried out at the University of Seville (Faculty of Medicine and Faculty of Nursing, Physiotherapy and Podiatry). The list of study sites is available in the trial registration information.

### Eligibility criteria {10}

Adults with a confirmed rheumatic diagnosis of RA for more than 2 years, affecting a minimum of one wrist, MCP, or PIP joints, will be assessed for eligibility. RA is defined following the diagnostic criteria of the American College of Rheumatology [4, 27]. Participants will have to show at least 4 out of 7 of the following signs or symptoms: (a) morning stiffness at or around hand joints and/or wrist for more than 1 h during 6 weeks or more; (b) three or more actively swollen joints during 6 weeks or more; (c) swollen wrist, MCP, or PIP joints; (d) asymmetrical joint swelling; (e) radiographic evidence of erosion or decalcification at hand or wrist; (f) presence of rheumatoid nodules; and (g) positive rheumatoid factor. Participants will have to self-report pain in their hands and/or wrists, and decrease ability to carry out daily life activities involving the hands [28]. In addition, participants must have a smartphone or tablet with internet access. Exclusion criteria are as follows: (a) previous history of hand surgery or fracture in the 6 months before data collection [28], (b) waiting list for upper limb surgery [20], (c) having received intramuscular or intraarticular steroid injections within 4 weeks [23], (d) pregnancy [2], and (e) a moderate or severe cognitive impairment [9]. Participants will be told not to be involved in other manual therapy interventions during the study period.

### Who will take informed consent? {26a}

A researcher team member will obtain verbal and written informed consents from all patients during the baseline assessment. Participants will be clearly informed of the study aims, and potential risks or benefits, and that they can withdraw the study at any point with no need to provide any specific reason for that. All participants will be assigned a study identification code linked to their personal information, which will be stored in a protected database accessible only to one researcher.

### Additional consent provisions for collection and use of participant data and biological specimens {26b}

On the consent form, participants will be asked if they agree to the use of their data should they choose to withdraw from the trial. This trial does not involve collecting biological specimens for storage.

## Interventions

### Explanation for the choice of comparators {6b}

The so-called conventional intervention is the standard treatment protocol used in the Andalusian Public Health System for self-management in patients with hand RA. The exercises are listed in a paper sheet and consist of upper limb stretching and strengthening exercises, with greater focus on hand joints and wrist.

### Intervention description {11a}

Participants assigned to the control group will receive the usual care provided by the Andalusian Public Health System in Primary Care during 3 months. First, the principal investigator will conduct an educational session explaining the intervention details and including motivational messages to encourage participants to carry out their treatment protocol. Then, the exercise program and recommendations are delivered in a paper sheet. Pictures of mobility and stretching exercises are present in this paper, focused on fingers, hand, wrist, and elbow joints. A written explanation of how to perform the exercises, and the number of sets and repetitions are included. Patients will carry out the protocol four times per week during 15–20 min each session.

**Fig. 2:**
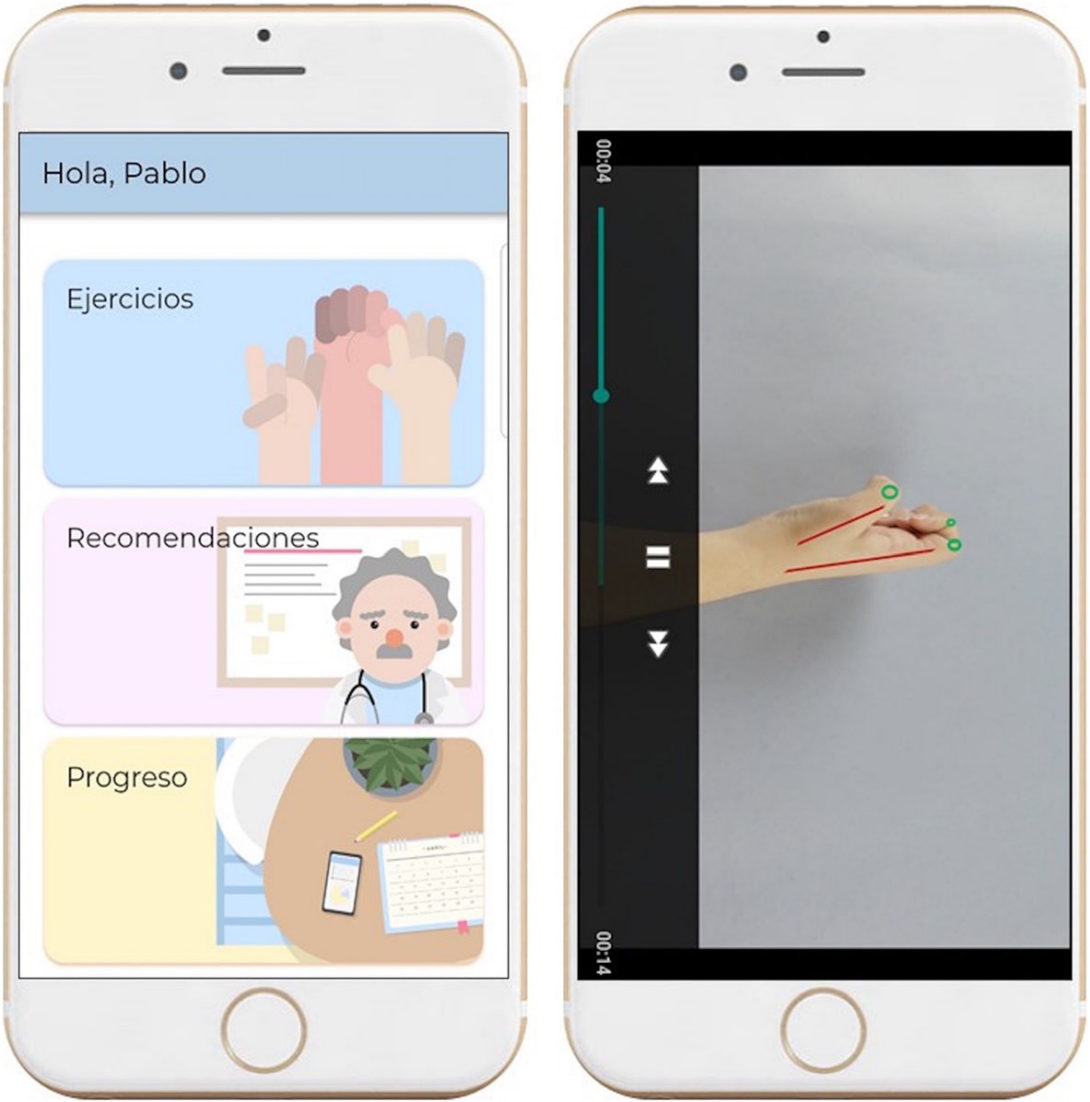
The CareHand application

Participants randomly assigned to the experimental group will use the CareHand app. First, the principal investigator will give an educational session explaining how to manage the CareHand app and its main features, along with a motivational speech to encourage participants. Then, each individual will receive a unique code and password to log into the app and to start working with their smartphones. The CareHand app includes an exercise protocol focused on mobility, stretching, and strengthening of the hands, as well as educational advices, and strategies to promote self-management. Exercises included are automatically adapted on a daily basis according to the patient condition and evolution, and are provided with a detailed explanation included in the application. Pain intensity using a Visual Analogue Scale (VAS) will be assessed before and after the exercise program, and other self-reported outcome questionnaires will be collected once a week with the app. Participants will be asked to use the app four times per week, during 15–20 min each session, for 3 months. The app also includes information related to the disease; self-management strategies for pain, fatigue, and daily live activities; dietary advices; and benefits of performing regular physical activity. The CareHand app records the adherence to treatment, which will be automatically sent to a research member by e-mail. Figure 2 includes several images of the app main menu and an example of one of the exercises listed in the app.

Participants in both groups will receive a follow-up phone call from a research collaborator (RC) at weeks 1 (t1), 3 (t2), 5 (t4), 9 (t5), and 13 (t6) to promote adherence and to solve any problems or doubts that participants may have. Outcome measures will be collected at baseline (t0), and at 1 month (t3) and 3 months (t7) from baseline. Data collection at 1 month (t3) will be conducted by a phone call, and the rest of the assessments will be carried out at the same public health center where participants were initially recruited.

### Criteria for discontinuing or modifying allocated interventions {11b}

Any possible participants’ requests for discontinuing with the intervention or the follow-up will be considered, and no explanations will be asked for. Participants will be also informed that they cannot be involved in a manual therapy treatment through the study process. Medical treatment modification or worsening disease will not be a reason to discontinue the intervention and the follow-up, but all this information will be registered.

### Strategies to improve adherence to interventions {11c}

Every participant will receive a follow-up phone call from a research member at weeks 1 (t1), 3 (t2), 5 (t4), 9 (t5), and 13 (t6) to control for treatment adherence. In addition, the exercise program will be graphically presented in a paper sheet (control group), or using audio-visual methods (CareHand group) with explanations to encourage adherence to treatment. Participants using the CareHand app will have access to relevant information, previously described, e.g., self-management strategies to deal with pain and fatigue and enhance performance in daily live activities, and dietary advices, among others, to encourage participants to continue with the proposed protocol.

### Relevant concomitant care permitted or prohibited during the trial {11d}

Participants will not be allowed to receive other manual therapy interventions or participate in other trials during the study period.

Medical stable treatment, using DMARDs, will be permitted throughout the study period. Pharmacological management of RA with DMARDs is very common, and its potential effects may increase when combined with exercise programs [20].

### Provisions for post-trial care {30}

Hand exercise programs are safe. In patients with RA, no adverse effects have been attributed to self-management with hand exercises [16]. Hence, no harm or adverse effects are expected from trial participation. Nonetheless, participants will be asked to report any possible side adverse effect.

### Outcomes {12}

#### Primary outcomes

The primary outcome is hand performance in different domains, as assessed with the Michigan Hand Outcome Questionnaire (MHQ). The MHQ is a well-established and commonly used tool for patients with RA and consists of six subscales that evaluate overall hand function, daily life activities, work performance, self-reported pain, esthetics, and satisfaction [29]. The final score ranges from 0 to 100, with higher scores indicating better performance, except for the pain domain, where higher scores represent more pain [30]. The MHQ has proven to be valid, reliable, and sensitive for people with hand RA, with good test-retest reliability (*r* = 0.66) and acceptable to excellent internal consistency (*α* = 0.66–0.90) [31]. In people with RA, the minimum clinically important difference (MCID) for the MHQ ranges from 3 (daily life activities) to 13 points (overall function) [32]. The validated Spanish version of the MHQ will be used in our study [33]. The MHQ will be collected at baseline (t0) and at 3 months (t7) from baseline.

#### Secondary outcomes

Secondary outcomes included self-reported pain intensity and morning stiffness, pain-free maximum grip and pinch force, and self-reported functional ability. Using a 0 to 10 VAS [34], where 0 denotes “no pain/no morning stiffness” and 10 denotes “the worst imaginable pain/morning stiffness,” participants will be told to report their average hand pain intensity and morning stiffness within the previous week. The VAS presents a good test-retest reliability (*r* = 0.94) [35] and is able to detect clinically relevant effects after intervention in people with different hand disorders [36]. The MCID for the VAS assessing pain intensity has been estimated at 1.1 points in patients with RA [37]. Self-reported pain intensity will be assessed at baseline (t0), and at 1 month (t3) and 3 months (t7) after baseline, whereas self-reported morning stiffness will be collected at baseline (t0), and at 3 months (t7) after baseline.

Handgrip strength is defined as the power of the hand muscles required in grasping or gripping, and can be divided in power grip (all the hand involved) or precision grip (only use of thumb and fingertips) [38]. A hydraulic hand dynamometer (Saehan SH5001, Saehan Corp., Masea, South Korea) will be used to evaluate power grip, following the guidelines of the American Society of Hand Therapy. The subject is seated, with the shoulder along the body and with no rotation, 90° elbow flexion, neutral forearm position, and the wrist with a “subtle” dorsal flexion (0–30°) [39]. Participants will be encouraged to grasp strongly without feeling pain or discomfort [39]. In patients with hand RA, a single trial is recommended to avoid discomfort and burden from subsequent measures [40]. The threshold for the MCID for power grip is estimated between 5 and 6 kg [41]. As regards precision grip, a pinch gauge (30 Lb. Mechanical Pinch Gauge, Baseline, USA) will be used. Following the same instructions, participants will be asked to hold the device between the index and middle fingers on one side and the thumb on the other side to evaluate the maximum pain-free pinch force [38]. Interrater reliability has shown to be almost perfect, with most ICC values over 0.97 [38]. A single measurement will be requested due to possible patient discomfort [42]. Handgrip and pinch strength will be assessed at baseline (t0) and at 3 months (t7) after baseline.

The self-reported functional ability will be evaluated by means of the shortened Disabilities of the Arm, Shoulder and Hand (DASH) questionnaire (*Quick* DASH). The *Quick* DASH questionnaire consists of 11 items scored on a 5-point Likert scale, resulting in a final score between 0 and 100 points, with higher values indicating more disability [43]. This tool includes two optional scales (work/music) which are not commonly collected [44]. The *Quick* DASH has shown to be highly reliable, internally consistent [45], and with good validity [46] and correlation with disease activity [43] in patients with RA. The test-retest and cross-sectional reliability and discriminant ability of this tool are similar to original DASH questionnaire [47]. The validated Spanish version of the questionnaire will be used [48]. The Quick DASH questionnaire will be collected at baseline (t0), and at 1 month (t3) and 3 months (t7) after baseline.

### Participant timeline {13}

**Fig. 3:**
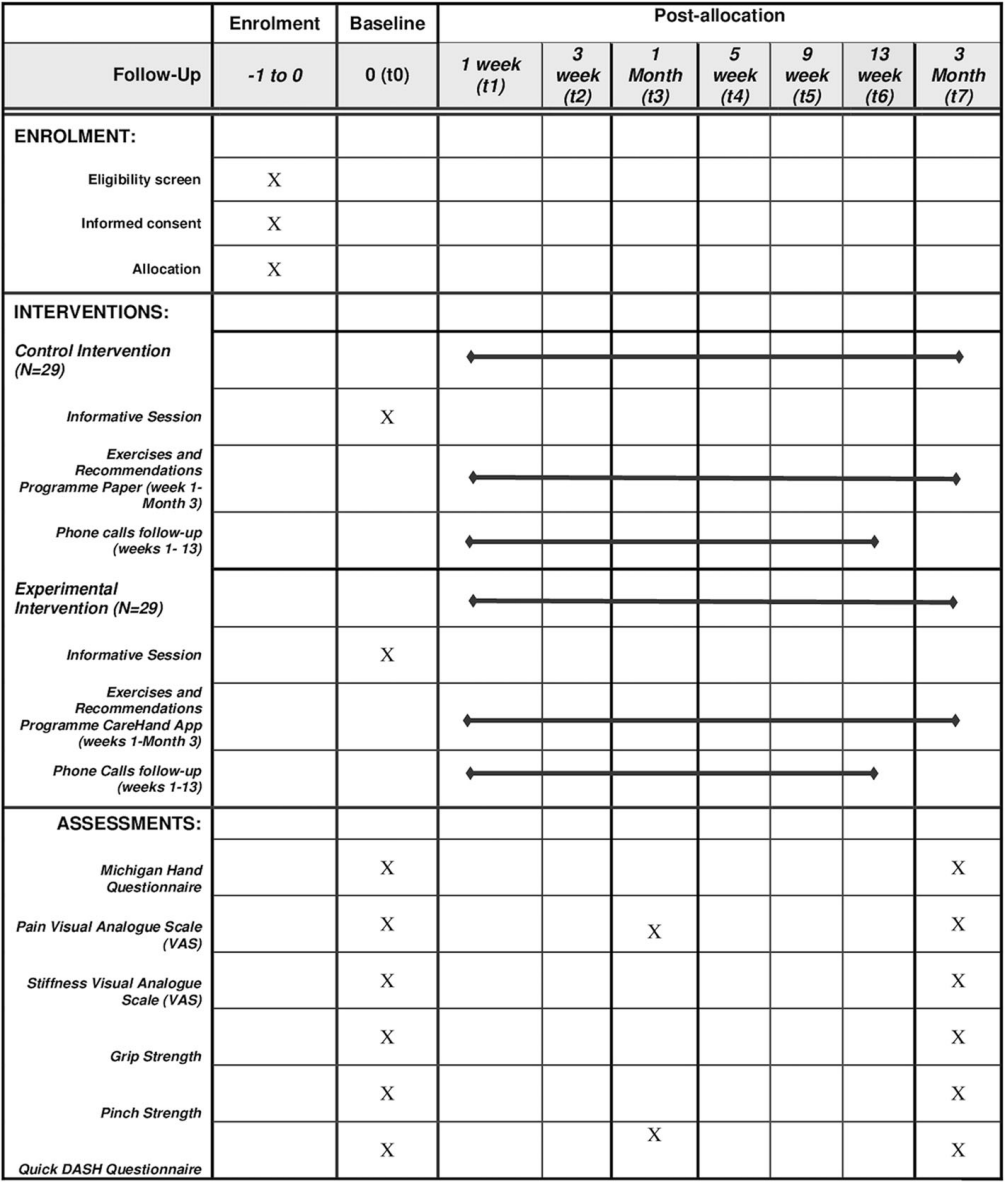
Standard Protocol Items Recommendation for Interventional Trials (SPIRIT) figure

A schematic diagram for participant timeline is shown in Fig. 3.

### Sample size {14}

Sample size estimation was based on detecting a mean between-group difference (CareHand vs. conventional) in the changes across the assessment points (baseline vs. after training, and baseline vs. 3-month follow-up) higher than 13 points for the overall MHQ, established as the clinically relevant threshold for this scale [49]. Assuming a two-tailed hypothesis, an alpha value of 0.05, and a desired power of 80%, 24 participants are needed per group to complete the study (Granmo Software, version 7.12, Institut Municipal d’Investigació Mèdica, Barcelona, Spain). To allow a 10% dropout rate, we will initially recruit at least 58 participants.

### Recruitment {15}

All possible eligible patients will be contacted directly via telephone to inform them about the aims and potential benefits of the study. Participants will be selected from the database of the Northern Seville and Aljarafe Health District, Andalusian Health Service, Seville, Spain. This Health District comprises 88 healthcare centers. For checking the recruitment viability, a query to this database was performed to confirm the number of patients diagnosed with RA. The search obtained 407 patients with hand RA as suitable for initial screening.

## Assignment of interventions: allocation

### Sequence generation {16a}

Using computer-generated random numbers in permuted blocks produced with an external website, all participants will be randomly assigned (1:1 ratio) to one of the study groups during baseline assessment, and after they have agreed to enroll in the study and signed the informed consent.

### Concealment mechanism {16b}

Sealed opaque envelopes will be used to conceal the treatment order allocation.

### Implementation {16c}

An external assistant will generate the random sequence. The principal investigator (PRSL) will enroll participants and randomly assign them to one of the study groups.

## Assignment of interventions: blinding

### Who will be blinded {17a}

Outcome assessors and data analysts will remain blinded to participants’ allocation group.

### Procedure for unblinding if needed {17b}

The study lead investigator, the researcher in charge of explaining and delivering interventions, and the participants will not be blinded to the group allocation.

## Data collection and management

### Plans for assessment and collection of outcomes {18a}

Assessors will participate in a training session where they will be instructed in each measurement tool and clinical questionnaire to be used. Any doubt will be resolved, and one training data collection session will be conducted. Due to the common discomfort of patients with hand RA, only one trial for strength measures will be performed [40].

All participants will be assessed three times during the study: at baseline, and at 1 month (t3) and 3 months (t7) from baseline. Baseline measurements (t0) will be completed before randomization. Assessment at 1 month will be carried out with a phone call, and data of self-reported pain intensity (VAS) and the Quick DASH questionnaire will be evaluated. Data collection at baseline and at 3-month follow-up will be conducted in the public healthcare centers and will involve all outcome measures.

The MHQ, as the primary outcome, is a reliable, valid, and sensible questionnaire, with acceptable to excellent internal consistency (*α* = 0.66–0.90), and good test-retest reliability (*r* = 0.66) [31]. Construct validity obtained the highest results between overall hand function and activities of daily living (*r* = 0.83), and between satisfaction, overall hand function, and activities of daily living (*r* = 0.81–0.83) [31]. In people with RA, the MCID for the overall hand function is at 13 points [32]. As regards secondary outcomes, the VAS is a valid tool to evaluate pain intensity in chronic conditions, with a good test-retest reliability (*r* = 0.94). The MCID for the VAS is established at 1.1 points in RA patients [37]. The use of a hand dynamometer to assess grip strength is common for people with upper limb chronic disorders. This tool shows high interrater reliability (ICC = 0.93) [41], with a clinically relevant threshold that ranges between 5 and 6 kg [39]. For pinch grip force, the proposed assessment protocol has shown to be excellent (ICC > 0.97) [39]. Finally, the Quick DASH is a valid, reliable, and consistent tool [45, 46], with a similar test-retest reliability and discriminant ability to that of the DASH questionnaire [47].

### Plans to promote participant retention and complete follow-up {18b}

Participants will receive a phone call from a research member at weeks 1 (t1), 3 (t2), 5 (t4), 9 (t5), and 13 (t6) for improving their participation and adherence to intervention. These phone calls are aimed to resolve doubts, problems, or misunderstandings with their intervention protocol and to encourage patients to continue working.

Additionally, participants using the CareHand app will benefit from additional methods to promote self-monitoring and self-management. The hand RA-related information included in the app may also help to encourage participants to complete the study, as this includes the possible benefits associated with regular physical activity.

### Data management {19}

Participants’ personal information will be assigned a unique study identification code that will be stored in a password-protected database accessible only to the principal investigator. Study data will be entered in a different computer and linked to the participants ID. A member of the researcher team will double-check the entered data for accuracy.

### Confidentiality {27}

Shared data between members of the research team will be included in a password-protected Excel file and only linked to the participants ID to respect confidentiality.

### Plans for collection, laboratory evaluation, and storage of biological specimens for genetic or molecular analysis in this trial/future use {33}

This trial does not involve collecting biological specimens for storage. Hence, there are no plans for collection, laboratory evaluation, or storage of biological specimens for genetic or molecular analysis during the present trial.

## Statistical methods

### Statistical methods for primary and secondary outcomes {20a}

The statistical processing will be performed with the PASW Advanced Statistics (SPSS Inc., Chicago, IL), version 24.0, and according to an intention-to-treat principle. Data will be reported as mean, standard deviation, and confidence intervals (95% CI) or in percentage frequencies. The Shapiro-Wilk test will evaluate the normal distribution of the variables. The between-group differences in the mean changes of the outcome measures after intervention will be calculated using repeated-measures mixed models with patients as random effect, and group (conventional or CareHand) and time (baseline, 1 month, and 3 months) as fixed effects, and with adjustments for baseline imbalance. The Spearman rank test or Pearson product-moment correlation coefficient analysis may be used to analyze the associations between clinical data and mean changes in the outcome measures. Statistical significance is set at a *p* value < 0.05.

### Interim analyses {21b}

There is no plan for any interim analysis or stopping data collection.

### Methods for additional analyses (e.g., subgroup analyses) {20b}

There are no planned subgroup analyses for any of the primary or secondary outcomes.

### Methods in analysis to handle protocol non-adherence and any statistical methods to handle missing data {20c}

This clinical trial aims to evaluate the effectiveness of this intervention under conditions as similar as possible to reality. Also, it will not be able to discard collateral effects of confounding or modifying variables such as concomitant treatments, abandonment, or partial non-adherence to intervention. Therefore, it has been decided to carry out an intention-to-treat analysis.

### Plans to give access to the full protocol, participant-level data, and statistical code {31c}

The study findings, full study report and protocol, will be made accessible after publication to all study participants, and the general public, upon request. There are no plans to grant public access to a de-identified dataset.

## Oversight and monitoring

### Composition of the coordinating center and trial steering committee {5d}

The investigator who conceived the study concept and design and procured funding (LGLR) will be in charge of the trial supervision, coordinating the entire project and reviewing the progress of the trial, and making corrections if needed.

### Composition of the data monitoring committee, its role, and reporting structure {21a}

In patients with RA, no adverse effects have been attributed to self-management with a home hand exercise program [16]. No adverse events, besides possible temporary minor soreness or discomfort, are expected. Hence, there will be no data monitoring committee. In case of any serious adverse events during the trial, the principal investigator will report them to Research Ethics Committee of University Hospitals Virgen del Rocio and Virgen Macarena, Seville, Spain.

### Adverse event reporting and harms {22}

Adverse events that may occur during the trial period will be self-reported by patients. The researchers supervising the interventions and those researchers collecting outcome measures at follow-up appointments will be also told to identify possible adverse events.

### Frequency and plans for auditing trial conduct {23}

The Public Foundation Progreso y Salud of the Health and Families Council, Andalusian Government, Spain, as the funding body, may audit some of their financed projects.

### Plans for communicating important protocol amendments to relevant parties (e.g., trial participants, ethical committees) {25}

No important protocol modifications are expected. If so, any changes will have to be reviewed by the Research Ethics Committee of Virgen del Rocio and Virgen Macarena University Hospitals, Seville, Spain.

## Dissemination plans {31a}

The study results, regardless of outcome, will be aimed to be published in international scientific journals, preferably an Open Access journal with a high impact, and throughout oral and written communications at national or international scientific congresses. In addition, social networks will be used to increase results dissemination.

## Discussion

The current project will evaluate the clinical effectiveness of a smartphone app combining hand exercise programs, education in RA-related information, and self-management strategies in patients with hand RA, when compared with a standard approach. We expect the most innovative approach to promote long-term positive effects on hand overall function, pain, stiffness, and strength. These positive outcomes may be applicable not only to patients with hand RA, but also to individuals with other chronic rheumatic diseases, such as osteoarthritis. The CareHand app is a new way to treat and monitor patients’ symptoms and evolution, using new technology that facilitates constant patient-therapist communication. Tele-rehabilitation and remote monitoring may promote a decreased RA activity status and facilitate physicians’ response in case of an exacerbated symptomatic period [50]. Therefore, this digital tool has the potential to generate a twofold positive impact on healthcare systems. First, it may help to reduce direct costs by lowering face-to-face consultations and by preventing unnecessary control visits [51], patient travel, and work leaves [52]. Second, it may improve the quality of the public health service offered to patients. In this sense, there is an urgent need to enhance the management provided in primary care for individuals with hand rheumatic conditions [53], because most of the interventions used in the clinical setting are not based on current evidence and clinical guidelines [52]. This gap between evidence-based and reported practice may lead to a large dissatisfaction in patients with RA [54], preventing some of them to attend to primary care even if they are severely affected and show functional difficulties [55, 56]. This may reflect the lack of awareness of available treatments and the key role of occupational and physical therapy in the management of this condition, with a low percentage of individuals referred to these health professionals (1 to 6%) [56], despite the positive effect of exercise protocols for hand RA [16].

In summary, the study findings could help to get a better understanding of how to deliver self-care strategies for people with a rheumatic condition, and to develop evidence-based knowledge about the impact of the use of new technologies in rheumatology and primary care practice in the public health system.

### Study limitations

One important study limitation may be the relatively short follow-up duration (3 months). Longer-term follow-ups may provide more information. Future research should consider this to understand participant’s adherence in the longer fashion. The natural course of RA usually involves remission and exacerbation periods, which may influence patient goals and evolution [57]. Therefore, recruitment and allocation should be performed carefully to account for this issue. Psychosocial factors associated with self-management [23] and chronic pain [58] will not be recorded in the study. Our main focus is on hand overall function, as suggested in recent systematic reviews on this topic [16]. Finally, the evaluation of the cost-effectiveness of the proposed interventions will not be conducted in the present study.

## Trial status

The trial is ongoing and is currently recruiting patients. Recruitment was initiated on March 1, 2020, and is expected to be completed by the end of November 2020. The trial is based on a protocol version of May 2018. The trial is registered with ClinicalTrials.gov (NCT04263974) on February 11, 2020. Last update was posted on April 15, 2020.

## Abbreviations

ANOVA: Analysis of variance
DASH: Disabilities of the Arm, Shoulder and Hand
DMARDs: Disease-modifying antirheumatic drugs
ICC: Intraclass correlation coefficient
MCID: Minimum clinically important difference
MCP: Metacarpophalangeal
MHQ: Michigan Hand Outcome Questionnaire
PIP: Proximal interphalangeal
RA: Rheumatoid arthritis
VAS: Visual Analogue Scale
